# Effect of gait speed on gait rhythmicity in Parkinson's disease: variability of stride time and swing time respond differently

**DOI:** 10.1186/1743-0003-2-23

**Published:** 2005-07-31

**Authors:** Silvi Frenkel-Toledo, Nir Giladi, Chava Peretz, Talia Herman, Leor Gruendlinger, Jeffrey M Hausdorff

**Affiliations:** 1 Movement Disorders Unit, Tel-Aviv Sourasky Medical Center, Tel-Aviv, Israel; 2 Department of Physical Therapy, Sackler School of Medicine, Tel-Aviv University, Israel; 3 Department of Neurology, Sackler School of Medicine, Tel-Aviv University, Israel; 4 Division on Aging, Harvard Medical School, Boston, MA, USA

**Keywords:** gait, speed, Parkinson's disease, treadmill, stride variability

## Abstract

**Background:**

The ability to maintain a steady gait rhythm is impaired in patients with Parkinson's disease (PD). This aspect of locomotor dyscontrol, which likely reflects impaired automaticity in PD, can be quantified by measuring the stride-to-stride variability of gait timing. Previous work has shown an increase in both the variability of the stride time and swing time in PD, but the origins of these changes are not fully understood. Patients with PD also generally walk with a reduced gait speed, a potential confounder of the observed changes in variability. The purpose of the present study was to examine the relationship between walking speed and gait variability.

**Methods:**

Stride time variability and swing time variability were measured in 36 patients with PD (Hoehn and Yahr stage 2–2.5) and 30 healthy controls who walked on a treadmill at four different speeds: 1) Comfortable walking speed (CWS), 2) 80% of CWS 3) 90% of CWS, and 4) 110% of CWS. In addition, we studied the effects of walking slowly on level ground, both with and without a walker.

**Results:**

Consistent with previous findings, increased variability of stride time and swing time was observed in the patients with PD in CWS, compared to controls. In both groups, there was a small but significant association between treadmill gait speed and stride time variability such that higher speeds were associated with lower (better) values of stride time variability (p = 0.0002). In contrast, swing time variability did not change in response to changes in gait speed. Similar results were observed with walking on level ground.

**Conclusion:**

The present results demonstrate that swing time variability is independent of gait speed, at least over the range studied, and therefore, that it may be used as a speed-independent marker of rhythmicity and gait steadiness. Since walking speed did not affect stride time variability and swing time variability in the same way, it appears that these two aspects of gait rhythmicity are not entirely controlled by the same mechanisms. The present findings also suggest that the increased gait variability in PD is disease-related, and not simply a consequence of bradykinesia.

## Introduction

Falls are one of the most serious complications of the gait disturbance in Parkinson's disease (PD) [1-7]. Beyond the acute trauma that they may cause, falls may lead to fear of falling, self-imposed restrictions in activities of daily living, and nursing home admission [1-6]. While traditional measures of gait and postural control do not adequately predict falls in PD [8], increased stride variability has been associated with an increased fall risk in older adults in general, as well as in patients with PD [9-13], suggesting that this aspect of gait may have clinical utility as an aid in fall risk assessment. More specifically, as a result of PD pathology, the ability to maintain a steady gait rhythm and a stable, steady walking pattern with minimal stride-to-stride changes is impaired in PD, i.e., stride variability is increased in PD [11,14-20].

The mechanisms underlying the increased stride variability in PD have not been widely investigated. The increased stride variability and impaired rhythmicity of gait in PD may reflect reduced automaticity and damaged locomotor synergies [15,16,21]. Indeed, external pacing and cues decrease stride variability in PD [20,22,23]. Levodopa therapy also reduces variability in PD, demonstrating the role dopaminergic pathways play in the impaired gait rhythmicity in PD [11]. Nonetheless, another possible explanation for the increased gait variability observed in PD is that it is simply a byproduct of bradykinesia and a lower gait speed, and not intrinsic to the disease. In addition to their effect on variability, levodopa and external cues also may increase gait speed in PD [11,24,25] and several studies suggest that stride variability increases if gait speed is lower than an optimal value [26,27]. Conversely, other reports indicate that walking speed and stride variability may be independent. No significant increase in stride time variability was observed in healthy elderly subjects even though they walked significantly slower than young adults [28,29]. Maki demonstrated that among older adults, variability was related to fall risk, while walking speed was related to fear of falling [13]. Miller et al observed a significant increase in gait speed, but no significant changes in variability measures after rhythmic training of PD subjects [30]. Hausdorff et al. found that gait variability measures were significantly increased in patients with Huntington's disease and patients with PD, compared to controls, whereas gait speed was significantly lower in PD, but not in Huntington's patients [16]. Thus, further work is needed to better understand the relationship between gait speed and stride variability in PD.

Previously, we described the effects of a treadmill on the gait of patients with PD at their comfortable walking speed [22]. Here we report on the influence of different walking speeds on the stride-to-stride variations in gait, specifically, stride time variability and swing time variability. The influence of speed was examined both in subjects with PD and in healthy controls to determine the degree to which any observed effects were specific to PD. We evaluated the effects of speed by studying subjects on a treadmill, where the speed could easily be fixed. In addition, subjects were tested while walking on level ground, both with and without the use of a walking aid.

## Methods

### Subjects

Thirty-six patients with idiopathic PD, as defined by the UK Brain Bank criteria [31], were recruited from the outpatient clinic of the Movement Disorders Unit at the Tel-Aviv Sourasky Medical Center. Patients were invited to participate if their disease stage was between 2 and 2.5 on the Hoehn and Yahr scale [32], if they did not experience motor response fluctuations, if they were able to ambulate independently, and if they did not use a treadmill for at least six months prior to the study. The PD patients were compared to thirty healthy control subjects of similar age who were recruited from the local community. Both PD and control subjects were excluded if they had clinically significant musculo-skeletal disease, cardio-vascular disease, respiratory disease, uncontrolled hypertension, diabetes or symptomatic peripheral vascular disease, other neurological disease (or PD in the case of the controls), dementia according to DSM IV criteria and MMSE, major depression according to DSM IV criteria, or uncorrected visual disturbances. The study was approved by the Human Studies Committee of Tel-Aviv Sourasky Medical Center. All subjects gave their written informed consent according to the declaration of Helsinki prior to entering the study.

The study population was characterized with respect to age, gender, height, weight, Mini-Mental State Exam (MMSE) scores [33] (a gross measure of cognitive function widely used to screen for dementia), and the Timed Up and Go test (TUG) (a gross measure of balance and lower extremity function) [34-37]. Subjects were also asked about their history of falls in the past year. The Unified Parkinson's Disease Rating Scale [38] (UPDRS) was used to quantify disease severity and extra-pyramidal signs in the subjects with PD.

### Protocol

After providing informed consent, subjects were familiarized with walking on a 35 meter walkway and walking on a motorized treadmill (Woodway LOKO System^®^, Germany). Subjects were tested four times on the walkway and four times on the treadmill at different speeds. Each test lasted two minutes. On level ground (the walkway), subjects were tested under four conditions in the following order: a) at their comfortable walking speed (CWS), b) at a self-selected slow speed, i.e., specifically, subjects were asked to walk at about 20% less than their CWS, c) at their self-selected CWS while using a walker (four rolling wheels, Provo Rolator, Premis Inc., Holland), and d) at a self-selected slow speed while using the walker (i.e. at 20% less than the CWS with the walker). On the treadmill, subjects were studied at four treadmill speeds: 1) the CWS observed when using a walker on the level walkway; 2) 80% of this CWS; 4) 90% of this CWS; and 4) 110% of this CWS. The order of the walking conditions on the treadmill was randomized.

Average gait speed on level ground was determined using a stopwatch by measuring the average time the subject walked the middle 10 meters of the 35 meter walkway during the two minutes of testing. Under all walking conditions, subjects walked with a safety harness around the waist that was attached only during the treadmill walking. Subjects walked on the treadmill with full weight bearing. Because the subjects walked while holding on to the handrails (of the walker or treadmill), the gait speed under condition (1), i.e., comfortable walking on the treadmill, was set to the gait speed under condition (c).

Initially, subjects walked up and down the 35 meters walkway to become familiar with the testing conditions. Before testing on the treadmill, subjects were given time to walk on the treadmill. This familiarization period was completed when the subject reported feeling comfortable walking on the treadmill at his or her preferred gait speed. Afterwards, subjects were given 5 minutes of rest to minimize any fatigue effects. Measurements on the treadmill were taken after about 30 seconds of gradually increasing the treadmill speed to the desired speed i.e., data collection was started only after subjects had reached a steady pace.

### Apparatus

A previously described computerized force-sensitive system was used to quantify gait and stride-to-stride variability [22,39]. The system measures the forces underneath the foot as a function of time. The system consists of a pair of shoes and a recording unit. Each shoe contains 8 load sensors that cover the surface of the sole and measure the vertical forces under the foot. The recording unit (19 × 14 × 4.5 cm; 1.5 kg) is carried on the waist. Plantar pressures under each foot are recorded at a rate of 100 Hz. Measurements are stored in a memory card during the walk and, after the walk, are transferred to a personal computer for further analysis. The following gait parameters were determined from the force record using previously described methods [9-11,17,22]: average stride time, swing time (%), stride time variability, and swing time (%) variability. Average stride length was calculated by multiplying the average gait speed by the average stride time. Variability measures were quantified using the coefficient of variation, e.g., stride time variability = 100 × (standard deviation of stride time)/(average stride time). Because values between the left and right feet were significantly correlated, we report here only the values based on the right foot.

### Statistical Analysis

Descriptive statistics are reported as mean ± SD. We used the Student's t and Chi-square tests to compare the PD and control subjects with respect to different background characteristics (e.g., age, gender). To evaluate the effect of speed on gait parameters and to compare the groups, we used Mixed Effects Models for repeated measures. For each gait parameter, a separate model was applied. The dependent variable was the gait parameter and the independent variables were the group (PD patients or controls), the walking condition (e.g., treadmill or walker), walking speed, and the interaction term group × walking condition × walking speed. P values reported are based on two-sided comparison. A p-value = 0.05 was considered statistically significant. All statistical analyses were performed using SPSS 11.5 and SAS 8.2 (Proc Mixed).

## Results

### Subject Characteristics

Demographic, anthropometric, and clinical characteristics of the patient and control groups are summarized in Table 1. Both groups were similar with respect to age, gender, height, weight, and the MMSE. Among the PD subjects, 63.9% were men; 60% of the controls were men (p = 0.746). As expected, subjects with PD took longer to perform the Timed Up and Go test. In terms of PD characteristics, the mean Hoehn and Yahr stage of the patients was 2.1 ± 0.2. The average score on the UPDRS (total) was 36.1 ± 11.5 and scores on Part I (mental), Part II (activities of daily living) and Part III (motor) were 2.2 ± 1.5, 10.5 ± 4.2, and 23.4 ± 7.4, respectively. On level ground, while using the walker, patients with PD walked more slowly and with increased variability of the stride time and swing time, compared to controls (see Table 1).

**Table 1:** Characteristics of the study population*

	**PD Subjects (n = 36)**	**Control Subjects (n = 30)**	**P-value**
**Age (yrs)**	61.2 ± 9.0	57.7 ± 7.0	0.078
**Height (m)**	1.68 ± 0.07	1.68 ± 0.09	0.914
**Weight (kg)**	73.75 ± 11.84	74.31 ± 12.52	0.855
**TUG test (sec)**	11.1 ± 1.9	9.7 ± 1.6	0.002
**MMSE**	27.9 ± 1.2	27.9 ± 1.9	0.919
			
**Average gait speed (m/sec)**	1.05 ± 0.14	1.21 ± 0.19	<0.001
**Average Stride Length (m)**	1.20 ± 0.14	1.33 ± 0.11	<0.001
**Average Stride Time (sec)**	1.15 ± 0.09	1.10 ± 0.10	0.222
**Average Swing Time (%)**	34.21 ± 2.85	35.37 ± 2.18	0.028
**Stride Time Variability (%)**	2.40 ± 0.61	1.87 ± 0.36	0.037
**Swing Time Variability (%)**	3.26 ± 1.35	2.63 ± 1.70	0.019

TUG: Timed Up and Go Test; MMSE: Mini Mental State Examination; Gait measures are taken from walking on level ground with a walker. Similar group differences were observed without the walker and on the treadmill.

### Effects of gait speed on level ground

Table 2 summarizes the effects of walking at a self-selected slow speed on gait on level ground. When asked to walk at a slow speed, the patients and the controls significantly reduced their gait speed (p < 0.001), by 17% and 15% when walking without a walker, respectively, and by 16% and 17% when walking with a walker, respectively. At the lower gait speed, both in the patients with PD and in the controls, the average stride length was significantly reduced and the average stride time and stride time variability were significantly increased. In contrast, swing time variability was not significantly changed when subjects walked at slower gait speeds. For all measures, among the patients with PD, the changes in gait that were made in response to the slower walking speed paralleled the changes made in the control subjects (i.e., there were no significant Group × Walking Condition × Speed interactions on level ground, p = 0.092 for stride time variability and p > 0.445 for all other measures).

**Table 2:** Effects of gait speed on spatio-temporal characteristics of gait in PD patients and controls on level ground

	Walking on ground		Walking on ground with a walker	
	Comfortable Walking Speed (CWS)	Slow Walking Speed (P value*)	Comfortable Walking Speed	Slow Walking Speed (P value*)

**a) PD subjects (n = 36)**				
Average gait speed (m/sec)	1.12 ± 0.15	0.93 ± 0.14 (<0.001)	1.05 ± 0.14	0.89 ± 0.12 (<0.001)
Average Stride Length (m)	1.25 ± 0.16	1.16 ± 0.14 (<0.001)	1.20 ± 0.14	1.12 ± 0.13 (<0.001)
Average Stride Time (sec)	1.12 ± 0.07	1.26 ± 0.11 (<0.001)	1.15 ± 0.09	1.27 ± 0.12 (<0.001)
Average Swing Time (%)	34.45 ± 2.60	33.78 ± 2.71 (0.006)	34.21 ± 2.85	33.78 ± 2.75 (0.054)
Stride Time Variability (%)	2.24 ± 0.74	3.03 ± 1.05 (<0.001)	2.40 ± 0.61	2.92 ± 1.31 (<0.001)
Swing Time Variability (%)	3.27 ± 1.25	3.57 ± 1.30 (0.164)	3.26 ± 1.35	3.41 ± 1.95 (0.456)
**b) Healthy Controls (n = 30)**				
Average gait speed (m/sec)	1.24 ± 0.18	1.05 ± 0.17 (<0.001)	1.21 ± 0.19	1.01 ± 0.19 (<0.001)
Average Stride Length (m)	1.33 ± 0.11	1.24 ± 0.10 (<0.001)	1.33 ± 0.11	1.23 ± 0.12 (<0.001)
Average Stride Time (sec)	1.08 ± 0.09	1.20 ± 0.13 (<0.001)	1.10 ± 0.10	1.25 ± 0.16 (<0.001)
Average Swing Time (%)	35.27 ± 1.97	34.79 ± 1.66 (0.093)	35.37 ± 2.18	34.91 ± 1.65 (0.113)
Stride Time Variability (%)	1.94 ± 0.36	2.38 ± 0.76 (0.003)	1.87 ± 0.36	2.65 ± 0.77 (<0.001)
Swing Time Variability (%)	2.80 ± 1.99	2.93 ± 1.36 (0.565)	2.63 ± 1.70	2.61 ± 1.47 (0.952)

*P values determined using a repeated measures approach (see Methods) based on comparisons between CWS walking to slower walking in PD and controls.

### Effects of gait speed on the treadmill

Table 3 summarizes the effects of treadmill speed on gait. On the treadmill, the effects were generally similar to those observed on level ground. Both in the patients with PD and in the controls, the average stride length and the average swing time were significantly reduced at the slowest treadmill speed (80% of CWS) and increased at 110% CWS. Average stride time was increased at the slowest treadmill speed and reduced at 110% of CWS. Stride time variability was significantly increased at 80% of CWS in the patients with PD.

**Table 3:** Effects of gait speed on spatio-temporal characteristics of gait in PD and controls on a motorized treadmill

	80% of CWS (P value*)	90% of CWS (P value*)	CWS	110% of CWS (P value*)
**a) PD Subjects (n = 36)**				
Average gait speed (m/sec)	0.84 ± 0.11 (<0.001)	0.95 ± 0.13 (<0.001)	1.05 ± 0.14	1.16 ± 0.16 (<0.001)
Average Stride Length (m)	1.05 ± 0.16 (<0.001)	1.13 ± 0.15 (<0.001)	1.20 ± 0.15	1.26 ± 0.14 (<0.001)
Average Stride Time (sec)	1.26 ± 0.15 (<0.001)	1.20 ± 0.13 (<0.001)	1.14 ± 0.11	1.09 ± 0.10 (0.020)
Average Swing Time (%)	32.39 ± 3.06 (<0.001)	33.02 ± 2.78 (0.051)	33.62 ± 2.48	33.89 ± 2.64 (0.032)
Stride Time Variability (%)	2.20 ± 1.55 (0.002)	2.01 ± 1.24 (0.062)	1.76 ± 0.57	1.61 ± 0.63 (0.826)
Swing Time Variability (%)	2.66 ± 1.57 (0.478)	2.55 ± 1.15 (0.839)	2.51 ± 0.98	2.48 ± 1.32 (0.855)
**b) Control Subjects (n = 30)**				
Average gait speed (m/sec)	0.97 ± 0.15 (<0.001)	1.09 ± 0.17 (<0.001)	1.21 ± 0.19	1.33 ± 0.21 (<0.001)
Average Stride Length (m)	1.19 ± 0.15 (<0.001)	1.25 ± 0.15 (<0.001)	1.33 ± 0.14	1.39 ± 0.14 (<0.001)
Average Stride Time (sec)	1.24 ± 0.15 (<0.001)	1.17 ± 0.14 (0.001)	1.11 ± 0.11	1.06 ± 0.10 (0.001)
Average Swing Time (%)	34.74 ± 1.65 (0.002)	35.12 ± 1.47 (0.074)	35.62 ± 1.45	36.25 ± 1.34 (0.026)
Stride Time Variability (%)	1.72 ± 0.74 (0.644)	1.56 ± 0.59 (0.597)	1.64 ± 0.80	1.44 ± 0.67 (0.178)
Swing Time Variability (%)	2.12 ± 0.92 (0.758)	1.99 ± 0.71 (0.424)	2.18 ± 1.22	2.00 ± 1.10 (0.459)

CWS: Comfortable walking speed as determined on level ground when walking with a walker.

*P values determined using a repeated measures approach (see Methods) based on comparisons between comfortable walking to slower/faster walking in PD and controls.

**Figure 1:**
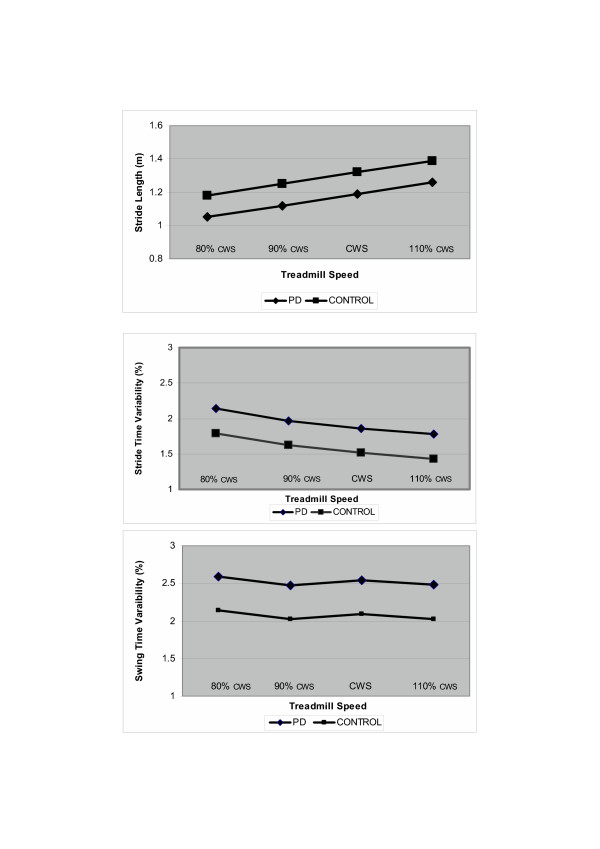
Stride length, stride time variability and swing time variability as measured at four different gait speeds on the treadmill. There were small but significant associations between gait speed and stride length and between gait speed and stride time variability, but swing time variability was not related to gait speed. CWS: comfortable walking speed. Values shown are based on mixed model estimates.

For all gait measures, the effects of the different walking speeds on treadmill were similar in the patients with PD and the control subjects (there was no significant Group × Slope interaction, p > 0.172). As can be discerned from the examples shown in Figure 1, all gait measures responded to the changes in speed in a more or less parallel fashion in the two groups. In both groups, there was a significant linear relationship between gait speed and average stride time (p < 0.0001), stride time variability (p = 0.0002), average swing time (p < 0.0001), and stride length (p < 0.0001). Note that while a significant relationship existed between speed and other measures, the changes with speed were, nonetheless, relatively small (see Table 3 and Figure 1). In both groups, swing time variability was not related to gait speed (p > 0.451).

## Discussion

Consistent with previous studies, we find a reduced stride length and average swing time, and an increased stride time variability and swing time variability in patients with PD [11,14-20]. The key findings of the present study are the relationships between gait speed and these measures. Stride length, stride time, swing time, and stride time variability were related to gait speed, both on level ground and on the treadmill, most notably at the slowest speeds, while swing time variability was independent of gait speed. Similar relationships were observed in the patients with PD and in the controls.

Yamasaki et al described a U-shaped relationship between stride length variability and gait speed when healthy subjects walked on a treadmill [26]. Minimum values were obtained at the CWS and increased when subjects walked slower or faster than the CWS. Similar U-shaped relationships in stride time variability and stride length variability have also been reported by others [27,40,41]. Yamasaki et al. suggested that minimal variability of stride length occurs at the CWS because, mechanically, the most efficient gait occurs at this speed and metabolic energy expenditures are at a minimum. Studies of mechanical and energetic expenditures on the treadmill support this explanation [42,43]. In the present study, we observed a linear relationship between gait speed and stride time variability and not a U-shaped relationship. The range of walking speeds tested may explain this apparent contradiction between previous studies. The linear trend that we observed for stride time variability may reflect one arm of the U-shape. Differences in study populations may also play a role here. Most of the previous investigations that examined the relationship between variability and gait speed studied healthy young adults. The present study focused on patients with PD and older adults. Mechanical and energy expenditure optimizations may be affected by aging and disease [44]. Interestingly, in a study of young and older adults, Grabiner et al [45] reported that gait speed did not affect the variability of walking velocity, stride length or stride time. To our knowledge, the present study is the first to examine the influence of speed on swing time variability. If the present results are confirmed, then it appears as if swing time variability may be used as a speed-independent marker of steadiness and fall risk. Nonetheless, future studies should evaluate the relationship between variability and gait speed over a wider range of speeds and perhaps also in young and older adults.

In previous studies that quantified stride time variability and swing time variability, these two measures were typically affected by disease and aging to similar degrees [9,16,46]. While both measures were different in PD and controls, walking speed affected stride time variability, but not swing time (%) variability in the present study. More than 20 years ago, Gabell and Nayak speculated about the differences between these two measures of variability [28]. They suggested that stride time variability is determined predominantly by the gait-patterning mechanism (repeated sequential contraction and relaxation of muscle groups resulting in walking), whereas swing time (double support time) variability is determined predominantly by balance-control mechanisms. Maybe because stride time variability reflects automatic rhythmic stepping mechanisms, it is more sensitive to different rhythmic rates, and hence walking speeds. Other studies have also observed that measures of gait variability may, at times, show independent behavior [45,47]. Additional biomechanical studies are needed to better understand the differences between stride time variability and swing time variability and the factors that contribute to each.

While more studies are needed to further clarify the relationship between gait speed and variability, the present findings support two conclusions. First, dysrhythmicity in gait in PD is caused by disease-related pathology. Stride time variability is influenced to a small degree by gait speed, but a close look at Table 3 suggests that the increased variability in PD is not simply the result of a reduced walking speed. The increased swing time variability in PD is apparently independent of gait speed. Furthermore, even when patients with PD walk at the same speed as controls (i.e., 90% of CWS in controls ≈ 100% of CWS in PD), swing time variability is increased in PD. Second, when studying gait variability, one should try to control for and take into account gait speed, perhaps by dictating the gait speed with a treadmill. When this is not possible, study of swing time variability may provide a marker of dysrhythmicity and instability that is independent of gait speed.

## Conflict of interest statement

The author(s) declare that they have no competing interests.

## Authors' contributions

SFT, NG, and JMH designed the study. SFT and TH participated in data collection. CP, JMH and LG performed the data analysis. SFT and JMH drafted the manuscript. All authors helped with the interpretation of the results, reviewed the manuscript, and participated in the editing of the final version of the manuscript.
